# Assessing the external validity of a randomized controlled trial of anthelminthics in mothers and their children in Entebbe, Uganda

**DOI:** 10.1186/1745-6215-15-310

**Published:** 2014-08-06

**Authors:** James D Millard, Lawrence Muhangi, Moses Sewankambo, Juliet Ndibazza, Alison M Elliott, Emily L Webb

**Affiliations:** Department of Global Health, Division of Clinical Medicine, Brighton and Sussex Medical School, Falmer, Brighton BN1 9PX UK; Medical Research Council, Uganda Virus Research Institute, PO Box 49, Entebbe, Uganda; London School of Hygiene and Tropical Medicine, Keppel Street, London, WC1E 7HT UK

**Keywords:** Helminths, Anthelminthics, External validity, Generalizability, Cluster sample community survey, Uganda

## Abstract

**Background:**

The ‘external validity’ of randomized controlled trials is an important measure of quality, but is often not formally assessed. Trials concerning mass drug administration for helminth control are likely to guide public health policy and careful interpretation of their context is needed. We aimed to determine how representative participants in one such trial were of their community. We explore implications for trial interpretation and resulting public health recommendations.

**Methods:**

The trial assessed was the Entebbe Mother and Baby Study (EMaBS), a trial of anthelminthic treatment during pregnancy and early childhood. In a novel approach for assessing external validity, we conducted a two-stage cluster sample community survey within the trial catchment area and compared characteristics of potentially-eligible community children with characteristics of children participating in the trial.

**Results:**

A total of 173 children aged three to five-years-old were surveyed from 480 households. Of children surveyed, we estimated that mothers of 60% would have been eligible for recruitment, and of these, 31% had actually been enrolled. Children surveyed were compared to 199 trial children in the same age group reviewed at annual trial visits during the same time period. There were significant differences in ethnicity between the trial participants and the community children, and in socioeconomic status, with those in the trial having, on average, more educated parents and higher maternal employment. Trial children were less likely to have barefoot exposure and more likely to use insecticide-treated bed nets. There were no significant differences in numbers of reported illness events over the last year.

**Conclusions:**

The trial had not enrolled all eligible participants, and those enrolled were of higher socioeconomic status, and had lower risk of exposure to the parasitic infections targeted by the trial interventions. It is possible the trial may have underestimated the absolute effects of anthelminthic treatment during pregnancy and early childhood, although the fact that there were no differences in reported incidence of common infectious diseases (one of the primary outcomes of EMaBS) between the two groups provides reassurance. Concurrent community surveys may be an effective way to test the external validity of trials.

**EMaBS Trial registration:**

ISRCTN32849447, registered 22 July 2005

**Electronic supplementary material:**

The online version of this article (doi:10.1186/1745-6215-15-310) contains supplementary material, which is available to authorized users.

## Background

The rise of evidence-based medicine has seen much emphasis placed on the internal validity of clinical trials but less attention has been given to external validity. Measures adopted for ensuring internal validity include the design of a trial which is appropriately powered to detect a clinically significant effect, the use of a control group selected by randomization and the ‘blinding’ of both participants and investigators to the intervention. Statistical analysis of the trial results then allows the classification of data on the basis of generally accepted levels of ‘significance’ [1, 2]. However, there have been increasing calls to take into account other factors when assessing the quality of evidence generated by trials [3–7]. These include biological plausibility, reproducibility and external validity [8]. External validity can be considered as the extent to which the results can be generalized to other circumstances. Whilst important, these factors may not have received the attention they deserve because their quality is not always easy to assess. However, failure to take these factors into account may limit a study’s usefulness. The implementation of findings that are not clearly applicable to the population in question has been called ‘evidence-biased medicine’ [9]. Increased awareness of external validity as a measure of study quality has led to its incorporation into several high-profile frameworks for the reporting and assessment of clinical trials [10–12]. The assessment of external validity is particularly important for trials in resource-poor settings, as these may be used to guide wide-ranging public health policy decisions, often in several settings or countries [13–15].

We aimed to assess the ‘external validity’ of the Entebbe Mother and Baby Study (ISRCTN32849447), a trial designed to investigate the effects of anthelminthic treatment in pregnancy and in childhood [16]. The primary outcomes included immunological responses to immunization and incidence of infectious and allergic disease in early childhood. This trial has now been reported and demonstrated a possible benefit of anthelminthic treatment during pregnancy for maternal anaemia, restricted to women with moderate to heavy hookworm infection, and a reduction in malaria incidence among children receiving quarterly anthelminthic treatment. However, there were none of the expected benefits for anaemia, birth weight, perinatal mortality, infant mortality or infant responses to immunizations. By contrast, there was an apparent adverse effect on infantile eczema [17–20].

There are relatively few studies which aim to assess the external validity of clinical trials. Most published studies focus on assessing the number of people included in the trial, as a proportion of those who would have been eligible for participation in the trial given the trial’s inclusion and exclusion criteria [21–27]. Other published studies rate trials on a scoring system devised for the purpose [28, 29], assess the adequacy of reporting of exclusion criteria [30, 31] or other generalizability measures [14], compare inferences derived from randomized controlled trial data with inferences derived from population-based studies addressing similar outcomes [32], compare outcomes between persons included and excluded from a trial [33, 34] or assess the representation of certain groups [35, 36]. Here, we report a novel approach to assess the external validity of a trial. Specifically, we conducted a community survey to assess whether participants in the Entebbe Mother and Baby Study were representative of the trial’s target population. By conducting a community-wide survey in which any appropriately aged child in the trial catchment area could potentially be enrolled, we hope to offer a more comprehensive assessment of external validity than studies to date. Our findings have implications for the generalizability of this trial, but also demonstrate an approach that may be of use in assessing the external validity of other trials.

## Methods

### Setting

The catchment area for the Entebbe Mother and Baby Study (EMaBS) was comprised of the Entebbe Municipality and Katabi sub-country, a peninsula on the northern shore of Lake Victoria, Uganda. Entebbe town is located approximately 40 km southwest of the capital, Kampala, has a population of approximately 90,500 and is the site of Uganda’s main international airport. Katabi sub-county borders Entebbe Municipality, has a population of approximately 59,000 and consists of semi-urban, rural and fishing communities. The EMaBS trial recruited pregnant women between April 2003 and November 2005. At the time of this investigation (between July and August 2008), EMaBS cohort children were aged three, four and five years. We therefore conducted a survey within the same catchment area, consisting of three, four and five-year-old children, both male and female.

### Study design

The community survey used a sampling strategy designed to reduce bias within a setting with limited prior demographic data. The study area comprised 47 administrative units known as wards. Census data detailing the number of households in each ward was available. A sample of 15 wards within the survey area was selected by random number generation, with probability of selection being proportional to the number of households. It was possible for one ward to be selected twice. Each ward was then mapped onto satellite imagery of the area with the help of locally available maps. Uninhabitable areas were excluded from mapping. The wards were divided into segments of equal geographical size (the same size across all wards) based on lines of latitude and longitude (degrees, minutes, seconds position format). These segments were then numbered and four segments from each ward were randomly selected using random number generation. The midpoint of each segment was identified by its coordinates and this was used as the starting point for sampling. The starting point was identified using a geographic information system (GIS) device (eTrex®, Garmin ™ Ltd, Kansas, United States) and the nearest house selected for sampling. Eight houses were then surveyed sequentially from this point, the next house to be sampled being the nearest to the previous house. A household was defined as a habitable roofed structure whose primary function was residence or, if used for dual purposes, had at least one active resident using the structure as their primary residence. In selected households that included a three, four or five-year old child, the parent or guardian was counselled and provided with written information in English and the vernacular of the area prior to obtaining written consent. If two or more eligible children lived in the same house, they were all surveyed if possible.

A questionnaire was then administered for each child. This was designed to match with data collected at the yearly trial visits undertaken by children enrolled in the Entebbe Mother and Baby Study, in order to obtain comparable information from both sources. In addition, during the period of the community survey supplementary information sheets were completed by trial participants during these yearly visits. These covered questions asked in the community survey but not routinely asked in the trial, or which had been asked in screening at enrolment into the trial, but were felt likely to have changed since that time.

#### Recruitment to EMaBS and community participation

EMaBS trial participants were recruited at the antenatal clinic at Entebbe Hospital over a two and a half year period. At the same time, the community was sensitized to the study. The mayor of Entebbe and sub-county chief of Katabi were informed and the research team visited all villages in the catchment area and held meetings with the local council (LC) leaders. LC leaders were asked to select community field workers, who were trained in simple data collection and subsequently followed up on participating children every two weeks until they were five-years-old. They met monthly and provided the main link between the research team and the community throughout the study period.

Inclusion criteria for the EMaBS trial required women to be resident in the study area, attending the Entebbe Hospital antenatal clinic and intending to give birth at the hospital, with no age limits. The exclusion criteria for the trial included not wishing to participate, not being willing to receive an HIV result, bloody diarrhoea, previous adverse reaction to anthelminthics or sulfadoxine-pyrimethamine (Fansidar™), already having a child in the trial, antenatal abnormalities, failure to complete screening or re-attend for enrolment, not being pregnant and anaemia (hemoglobin <8 g/dL).

#### Eligibility and uptake assessment

In the survey, we first aimed to assess what proportion of community children would have been potentially eligible for EMaBS trial participation. Children were deemed to have been potentially eligible if, at the time of the child’s birth, the mother was resident within the study area and attended the Entebbe Hospital antenatal clinic. We then aimed to estimate what proportion of potentially eligible children had actually been enrolled in the trial. We were unable to directly assess the impact of the other EMaBS inclusion and exclusion criteria.

### Outcomes

Socio-demographic characteristics: The outcomes assessed were primary carer/s, maternal and paternal age and health status, level of maternal and paternal education, maternal employment and wage, maternal tribe and other socioeconomic parameters (including housing materials, crowding, water and electricity provision). These factors are unlikely to have been affected by the trial intervention and therefore reflect inherent characteristics of the study population.

Disease risk factors and comorbidities: Outcomes assessed were barefoot exposure, lake exposure (both risk factors for helminth infection), mosquito net usage and insecticide-treatment of household nets (risk factors for malaria).

Diseases and anthropometry outcomes were as follows: we recorded self-reported disease episodes of malaria, diarrhoea, pneumonia, measles and tuberculosis. We did not ask directly about HIV because of the limitations placed on confidentiality in the field setting and concerns over the reliability of any results obtained. We also measured height, weight, mid to upper arm circumference and head circumference. These outcomes may have been influenced by the trial interventions.

### Statistical analysis

Data were analyzed using Stata version 12 (StataCorp, Texas, United States). Data from the youngest of each pair or group of siblings in the community survey were excluded from the analysis, in order to make the inclusion criteria comparable with EMaBS (where one of the exclusion criteria was having a child already in the trial). Clustering at the ward and segment level was taken into account in the analysis. Clustering at the household level was not allowed for in the analysis due to the small number of households remaining with multiple children after younger siblings were excluded from the analysis. The svy commands in Stata were used to allow for the clustering. Distributions of parental and child characteristics in the two groups were first compared using simple tabulations, with design-based Pearson’s F statistics calculated to test for differences in characteristics between the community survey and EMaBS children. Logistic regression was used to calculate crude and adjusted odds ratios (ORs) and associated 95% confidence intervals (CIs), allowing for the sampling design. Multivariable logistic regression analysis was conducted to control for potential confounding. A hierarchical approach was used to decide which variables should be treated as potential confounders in the multivariable analysis. Maternal and paternal sociodemographic parameters were considered as potential confounders for each other, for household characteristics, for disease risk factors and comorbidities, and for diseases and anthropometry. Household characteristics were considered as potential confounders for each other, for disease risk factors and comorbidities and for diseases and anthropometry. Finally, disease risk factors and comorbidities were considered as potential confounders for diseases and anthropometry.

### Ethical approval

Both EMaBS and the community survey received ethical approval from the Science and Ethics Committee of the Uganda Virus Research Institute (GC/127), Uganda National Council for Science and Technology (MV 625) and the London School of Hygiene and Tropical Medicine ethics committee (07/303).

## Results

**Figure 1:**
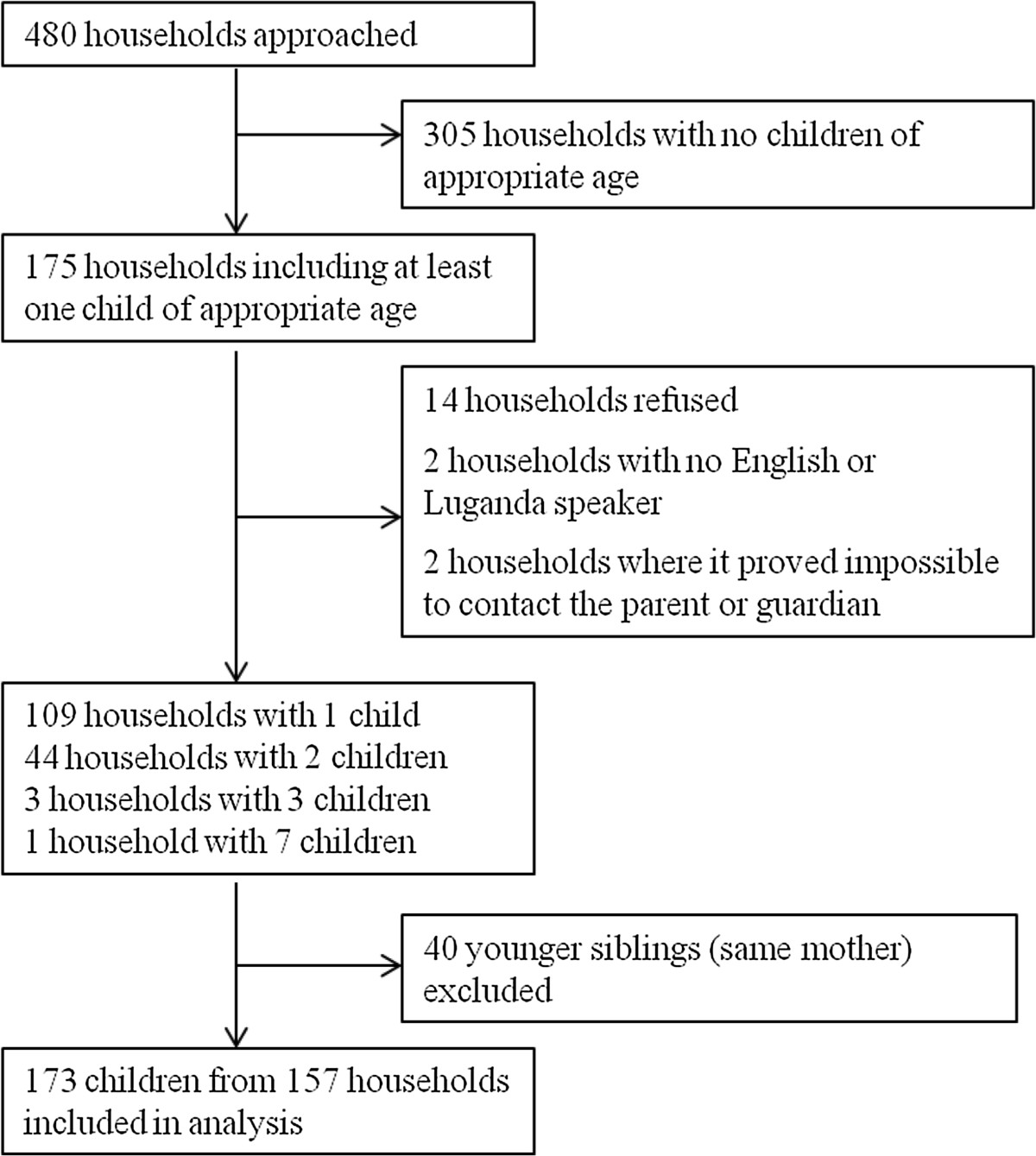
**Breakdown of households approached during the community survey and number of children in each.**

In total, 480 households were surveyed; eight households from each of four locations in 15 wards. The breakdown of these is provided in Figure 1. One hundred and seventy three children were eligible for inclusion in the analysis. During the survey period 199 trial children visited the trial clinic, of whom 128 completed supplementary information questionnaires. There were no differences in characteristics of those who completed supplementary questionnaires compared to those who did not.

Of the 173 children seen in the community, 104 (60%) had mothers who would have been potentially eligible for recruitment into the trial. Of the remaining children, 38 (55%) had mothers who were not resident in Entebbe or Katabi at the time of delivery, an additional 28 (41%) had mothers who did not receive antenatal care at Entebbe Hospital and were therefore not available for recruitment, and there was no information available on where mothers had received antenatal care for 3 (4%). Of the 104 children who were potentially eligible for inclusion, 32 (31%) were in the trial.

There were significant differences between the ethnic makeup of the two populations, based on the mother’s tribe. Of particular note only half the mothers in the trial were Buganda compared to 65% in the community, and being a member of the Banyankole, Batoro or Banyarwanda tribes was twice as common in the trial mothers (Table 1).

**Table 1:** **Comparison of characteristics of EMaBS trial annual visit children and community survey children**

	Trial annual visit (n = 199)	Community survey (n = 173)	OR (95% CI)^1^	***P*** ^1^(***P*** trend)
**Sociodemographic variables** ^**2**^				
Maternal tribe				
Baganda	99 (50%)	110 (65%)	1	0.05
Banyankole/Batoro/Banyarwanda	37 (19%)	17 (10%)	0.41 (0.20, 0.87)	
Basoga	11 (6%)	6 (4%)	0.49 (0.16, 1.50)	
Other	52 (27%)	37 (22%)	0.64 (0.34, 1.21)	
Maternal education (mv: 0, 13)				
None	8 (4%)	22 (14%)	4.43 (1.85, 10.57)	<0.001
Primary	103 (52%)	64 (40%)	1	(0.08)
Secondary	67 (34%)	68 (43%)	1.63 (0.97, 2.76)	
Tertiary	21 (11%)	6 (4%)	0.46 (0.17, 1.21)	
Paternal education (mv: 37, 38)				
None/primary	31 (19%)	46 (34%)	1	<0.001
Secondary	79 (49%)	75 (56%)	0.64 (0.36, 1.14)	(<0.001)
Tertiary	52 (32%)	14 (10%)	0.18 (0.09, 0.37)	
Maternal age (mv: 0, 12)	24.2 (4.7)	23.1 (5.6)	0.96 (0.92, 1.00)	0.04
Paternal age (mv: 121, 54)	34.8 (7.1)	33.8 (7.4)	0.98 (0.95, 1.02)	0.33
Maternal employment (mv: 73, 8)				
Unemployed	56 (44%)	123 (75%)	1	<0.001
Employed	70 (56%)	42 (25%)	0.27 (0.16, 0.46)	
Annual maternal income (Ugandan Shillings) (mv: 77, 1)				
Zero	67 (55%)	126 (73%)	1	0.002
0-50000	26 (21%)	15 (9%)	0.31 (0.15, 0.64)	(0.08)
50001-100000	23 (19%)	18 (10%)	0.42 (0.21, 0.83)	
100001-	6 (5%)	13 (8%)	1.15 (0.40, 3.29)	
**Household circumstances**				
Crowding (mv: 74, 1)				
<=3 people/room	77 (62%)	126 (73%)	1	
>3 people/room	48 (38%)	46 (27%)	0.59 (0.37, 0.93)	0.02
Roofing (mv: 74, 1)				
Iron/tiles	118 (94%)	169 (98%)	1	
Banana leaves/grass	7 (6%)	3 (2%)	0.30 (0.06, 1.56)	0.13
Walls (mv: 74, 1)				
Bricks	108 (86%)	164 (95%)	1	
Mud/metal	17 (14%)	8 (5%)	0.31 (0.10, 0.92)	0.03
Fuel source (mv: 73, 1)				
Firewood	25 (20%)	27 (16%)	1	0.04
Charcoal	89 (71%)	141 (82%)	1.47 (0.67, 3.22)	
Paraffin/gas/elec	12 (10%)	4 (2%)	0.31 (0.08, 1.16)	
Electricity supply (mv: 71, 1)				
Yes	60 (48%)	67 (39%)	1	
No	66 (52%)	105 (61%)	1.42 (0.81, 2.50)	0.21
Water source (mv: 71, 1)				
Lake/well/borehole	15 (12%)	14 (8%)	1	
Standpipe/domestic tap	111 (88%)	158 (92%)	1.53 (0.52, 4.51)	0.44
**Disease risk factors**				
Barefoot exposure (mv: 71, 6)				
Never/rarely	47 (37%)	7 (4%)	1	<0.001
Often	79 (63%)	160 (96%)	13.60 (5.55, 33.30)	
Lake exposure (mv: 71, 1)				
Never	108 (86%)	145 (84%)	1	
Ever	18 (14%)	27 (16%)	1.12 (0.59, 2.13)	0.74
Child sleeps under net (mv: 55, 1)				
Always	113 (78%)	88 (51%)	1	<0.001
Sometimes	13 (9%)	28 (16%)	2.77 (1.32, 5.79)	(<0.001)
Never	18 (13%)	56 (33%)	3.99 (2.11, 7.56)	
Household nets treated?				
None	56 (44%)	109 (94%)	1	<0.001
Some/all	71 (56%)	7 (6%)	0.05 (0.02, 0.12)	
**Reported disease episodes**				
Malaria (mv: 14, 1)				
No	88 (48%)	74 (43%)	1	
Yes	97 (52%)	98 (57%)	1.20 (0.78, 1.86)	0.41
Malaria (slide proven; mv: 44, 2)				
No	103 (66%)	111 (65%)	1	
Yes	52 (34%)	60 (35%)	1.07 (0.69, 1.65)	0.76
Diarrhoea (mv: 14, 2)				
No	120 (65%)	130 (76%)	1	
Yes	65 (35%)	41 (24%)	0.58 (0.35, 0.97)	0.04
Pneumonia (mv: 7, 4)				
No	188 (98%)	164 (97%)	1	
Yes	4 (2%)	5 (3%)	1.43 (0.38, 5.35)	0.59
Measles (mv: 7, 2)				
No	181 (94%)	147 (86%)	1	
Yes	11 (6%)	24 (14%)	2.69 (1.22, 5.92)	0.01
**Anthropometric measurements**	Mean (SD)	Mean (SD)		
Weight-for-age z-score	-0.46 (0.97)	-0.61 (1.04)	0.86 (0.68, 1.09)	0.21
Height-for-age z-score	-0.90 (0.96)	-0.91 (1.27)	0.99 (0.80, 1.24)	0.96

^1^Odds ratios use the trial population as the reference group and are adjusted for clustering.

2mv denotes number of individuals with missing values in EMaBS and Community Survey groups, respectively.

Levels of both maternal and paternal education were, on average, higher amongst trial children (Table 1). There was no significant difference in parental health or primary carer between the two groups (data not shown). In crude analysis mothers in the trial were, on average, significantly older than those in the community, but this difference was no longer significant after adjusting for tribe and education (Table 2). There was no significant difference in the mean paternal age. There were significant differences in maternal employment and income. Mothers of trial children were more than twice as likely to be employed, and subsequently tended to have a higher income, although income was no longer significantly different between the two groups once maternal employment was taken into account.

**Table 2:** **Characteristics showing differences between EMaBS children and community survey children after multivariable analysis**

Sociodemographic variables	Adjusted OR (95% CI)^1^	***P*** ^1^
Maternal tribe		
Muganda	1	0.03
Munyankole/Mutoro/Munyarwanda	0.31 (0.14, 0.71)	
Musoga	0.44 (0.14, 1.32)	
Other	0.76 (0.36, 1.61)	
Maternal education		
None	5.89 (2.28, 15.17)	<0.001
Primary	1	
Secondary	2.07 (1.21, 3.55)	
Tertiary	0.97 (0.31, 3.03)	
Paternal education		
None/primary	1	<0.001
Secondary	0.64 (0.33, 1.23)	
Tertiary	0.18 (0.08, 0.40)	
Maternal employment		
Unemployed	1	
Employed	0.21 (0.12, 0.37)	<0.001
Crowding		
<=3 people/room	1	
>3 people/room	0.54 (0.30, 0.97)	0.04
Walls		
Bricks	1	
Mud/metal	0.28 (0.11, 0.73)	0.01
Barefoot exposure		
Never/rarely	1	
Often	17.91 (6.82, 47.07)	<0.001
Child sleeps under net		
Always	1	0.02
Sometimes	2.09 (0.88, 4.98)	
Never	3.66 (1.42, 9.47)	
Household nets treated?		
None	1	
Some/all	0.07 (0.03, 0.17)	<0.001
Measles		
No	1	
Yes	0.23 (0.07, 0.80)	0.02

^1^Odds ratios use the trial population as the reference group and are adjusted for clustering. Results for tribe, maternal education and paternal education adjusted for each other; results for maternal employment adjusted for tribe and parental education; results for crowding and walls adjusted for tribe, education, employment and each other; results for barefoot exposure, child bed-net usage and household net treatment adjusted for all other factors except measles; results for measles adjusted for all other factors.

Trial children were more likely to live in houses with more than three people per room and more likely to live in houses with metal or mud walls than community survey children (Table 2). In crude analysis, there was a significant difference in fuel source between the two groups, but this was no longer significant after adjustment for other household characteristics (*P* = 0.33). There were no significant differences in roofing materials, electricity or water source between the two groups (Table 1).

There were several differences in disease risk factors between the community and trial children (Tables 1, 2). Reported mosquito net usage was markedly higher in the trial group, with an increased likelihood of the net being treated. Reported frequent barefoot exposure was higher in the community and lake exposure was similar in the two groups (Table 1). There was no significant difference between reported disease measures of malaria, slide-proven malaria or pneumonia (Table 1). Reported diarrhoea was more common among trial children, although this difference was not significant after adjusting for other parental and household factors (*P* = 0.26). Reported measles, although rare, was more common in community children (Table 2).

## Discussion

We have presented a community survey as a novel method for assessing aspects of the external validity of a randomized controlled trial. We found that EMaBS trial participants were on average, more likely to have parents with higher levels of education and who were in employment, more likely to come from non-local tribal groups, more likely to sleep under a bed-net and less likely to have barefoot exposure (a risk factor for helminths) than children in the target population for the trial. However, we found no differences in reported episodes of common childhood diseases between trial participants and children in the community survey. We estimate that 31% of eligible children in the community were enrolled in the trial, and although it was not possible to assess all trial exclusion criteria in the community survey, refusal and exclusion criteria are unlikely to account for the lack of enrolment of all the remaining children.

Under a fifth of children in the community had been enrolled in the trial. Approximately 22% of children were ineligible on the basis of non-residency at the time of birth, and these children may have differed in important ways from those who had not recently migrated. This is an unavoidable factor when considering the non-representativeness of trials to their community. It is possible that migration into a trial area, particularly if large and sustained, may account for a significant proportion of any non-representativeness and this highlights the need to interpret the results of trials carefully in the light of shifting demographic patterns. A similar proportion of children were ineligible because their mothers received antenatal care outside of the hospital setting, a group previously noted to differ from those who did receive hospital-based antenatal care in this area [37]. Our survey was not powered to assess whether those children excluded in this way were significantly different from those eligible for the trial. However, the relatively high level of hospital-based antenatal care is reassuring and provides support for the recruitment strategy used in this trial, hence lending support to its external validity.

**Figure 2:**
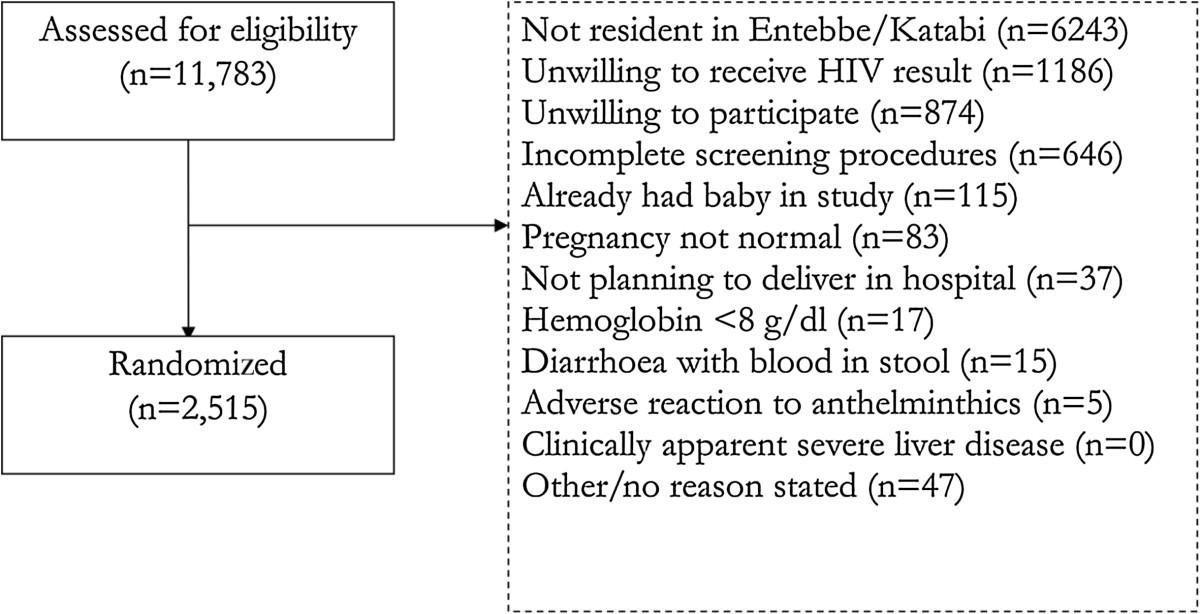
**Extract from CONSORT diagram for EMaBS showing number assessed for eligibility, numbers not enrolled (with reasons) and number randomized.**

Approximately 60% of mothers of children in the community survey would have been eligible for recruitment into EMaBS on the basis of residence and of antenatal care in Entebbe Hospital, and 31% of these were enrolled in the trial. Non-participation could have been secondary to refusal, exclusion on the basis of trial exclusion criteria or not having been approached for recruitment into the trial. In comparison, data from the EMaBS trial itself (Figure 2) shows that of 11,783 mothers initially assessed for inclusion in the trial, 5,388 (46%) were resident in and obtained antenatal care in Entebbe, planned on delivering in Entebbe hospital, and did not already have a baby in the trial. Of these, 2515 (47%) were subsequently enrolled [18]. However, the discrepancy between the figures of 31% and 47% implies a failure to fully assess all potentially eligible mothers at the antenatal clinic, most likely as a result of the heavy patient burden at the clinic. It is possible that this could have introduced bias due to differences in characteristics of women who would be able or willing to wait for the research procedures after receiving their standard antenatal care. This would be a potential threat to this trial’s external validity if a systematic bias occurred. Figure 2 also shows the reasons for which mothers were excluded from the EMaBS trial that could not be assessed in the community survey. It is possible that sociodemographic characteristics of women with these exclusion criteria differed from characteristics of those who did not meet them. If so then this could be an explanation for the different characteristics seen between the trial and community survey participants.

Data from the EMaBS trial (Figure 2) give an estimated eligibility fraction (proportion eligible of those screened) of 23% and an estimated enrolment fraction (proportion randomized of those eligible) of 98%, yielding a recruitment fraction of 22%. As far as we are aware, there is no clear consensus on an acceptable level of trial participation of eligible persons when reviewed at the community level, with most studies focusing on recruitment ratios at the level of the point of recruitment or primary care [22, 25–27]. It is therefore difficult to assess the uptake rate in this trial in context and this is an area in need of further study.

Whilst the majority maternal tribe in both groups was the local Baganda, the proportion within the trial participants was 15% less than within the community at large. The difference was largely made up by a higher proportion of mothers from the Banyankole and Luo tribes. The Banyankole are a Western Ugandan tribe and the Luo are from Northern Uganda [38]. The possibility of differences between the trial and community children, based on differences due to ethnic origin, therefore exists. Representation of minority ethnic groups in clinical trials is usually an issue of under-representation, with consequent difficulty extrapolating results to these groups [21, 35, 36, 39–41]. It is therefore reassuring that none of the minority tribes for this region were under-represented in the trial compared to the community.

Children enrolled in the trial were more likely to be from families of higher socioeconomic status. Maternal employment status and income were higher, as was the level of both maternal and paternal education. There was a difference in crowding status, with trial children more likely to live in crowded circumstances. It is suggested that increased crowding may be a function of increased wealth in some circumstances. In particular it may be the case that families with increased income remain in their current property, but members of extended family or friends may move into the residence. This is consistent with findings from other settings, where in general, trial participation is more likely amongst those of higher socioeconomic status [22, 24, 33, 35, 40]. These differences might have implications for the trial results because worm burden is directly related to poverty [42], and in this study population, the mother’s education was associated with a lower prevalence for every infection in mothers at enrolment [43]. Hence if, as seems possible, the helminth burden in the trial population was lower than that in the general community, the effect of the anthelminthic treatment intervention on trial outcomes would have been attenuated in the trial population compared to the theoretical effect of such an intervention applied to the whole community.

In general, disease exposure risks were fewer amongst the trial children with more bed-net use and less barefoot exposure. This difference persisted after adjustment for parental socioeconomic status. It may be the case that a systemic bias in selection for children in the trial resulted in selection of children with lower risk exposure status (not mediated by parental socioeconomic status). Alternatively, health education as part of the trial may have led to less exposure prone behaviour in the families of the trial children. However, importantly, with the exception of measles, there were no differences between the two groups in terms of reported episodes of infectious diseases, one of the EMaBS primary outcomes. This suggests that the sociodemographic differences we have observed between trial and community survey participants would not have led to biased intervention effect estimates on this primary outcome in the EMaBS trial.

### Strengths and limitations

We were unable to select participants at the level of the child due to the lack of sampling frame and therefore used a multi-stage sampling survey approach. This was done using a predefined random sampling approach and was taken into account in the analysis, however, it is possible that our sampling strategy will have over-represented children in less populated areas. There may be important differences between these children and children in more populous areas, hence potentially biasing our results. The fact that we used a random sampling approach should have reduced bias in the sample, however the clustered nature of our design means we may have misrepresented variables which are geographically confined. For example, there was one sampled area (a military barracks) which we were not permitted to enter, whereas children enrolled in the trial do live in this area. Indeed, this may explain the higher proportion of abodes constructed from metal amongst the trial children, as this is the predominant material used in military barracks in Entebbe.

The high response rate in the community survey means that selection bias should be minimized, and data completeness for the community survey was high. For the trial children we used several different sources of information, meaning the completeness of the data for each variable differed. In particular, a number of variables that were assessed from the supplementary forms for EMaBS children are limited because out of the 199 children assessed, only 128 supplementary forms were completed. There is no reason to believe this introduced bias since characteristics of the children who completed forms were similar to those of the children who did not, however, it limited the study’s power to detect differences between the groups for these variables. It is possible that responses to the questions may have been systematically different between the trial and community-surveyed groups. This may have occurred because the interviewers in the trial clinic and the community were different throughout. Also, the parents or guardians of the non-trial children may have responded to questions on recent childhood illnesses differently to the parents or guardians of the trial children, for instance, participation in the trial may have sensitized them to keeping a more accurate record of their child’s illnesses. We included trial children in our community survey (five children provided data to both the trial and community survey during the study period) and whilst this was intended in the survey design on the basis that they are part of their community and thus not as such a limitation, it could have led to a slight underestimation of the differences between the groups.

## Conclusions

Recruitment at the level of the antenatal clinic did not achieve enrolment of all eligible participants into this trial and this was unlikely to be fully explained by refusal or exclusion criteria. The study population was significantly different from the community at large on the basis of ethnic composition and socioeconomic status. There appeared to be increased disease risk factors in the community survey group but little difference in terms of reported disease episodes. To our knowledge, this is the first study of its kind using a community survey to assess the external validity of a randomized controlled trial. External validity is a very important component of the assessment of trials and this approach offers a cost-effective, practical and robust method of assessing the validity of a trial.

## Authors’ original submitted files for images

Below are the links to the authors’ original submitted files for images. Authors’ original file for figure 1 Authors’ original file for figure 2

## Abbreviations

CI: Confidence interval
EMaBS: Entebbe Mother and Baby Study
GIS: Geographic information system
HIV: Human immunodeficiency virus
km: Kilometer
OR: Odds ratio.
