# Supplementary figures and images for: Assessing the external validity of a randomized controlled trial of anthelminthics in mothers and their children in Entebbe, Uganda
Source: Trials. 2014 Aug 6;15:310. doi: 10.1186/1745-6215-15-310 (PMC4138365; doi:10.1186/1745-6215-15-310)

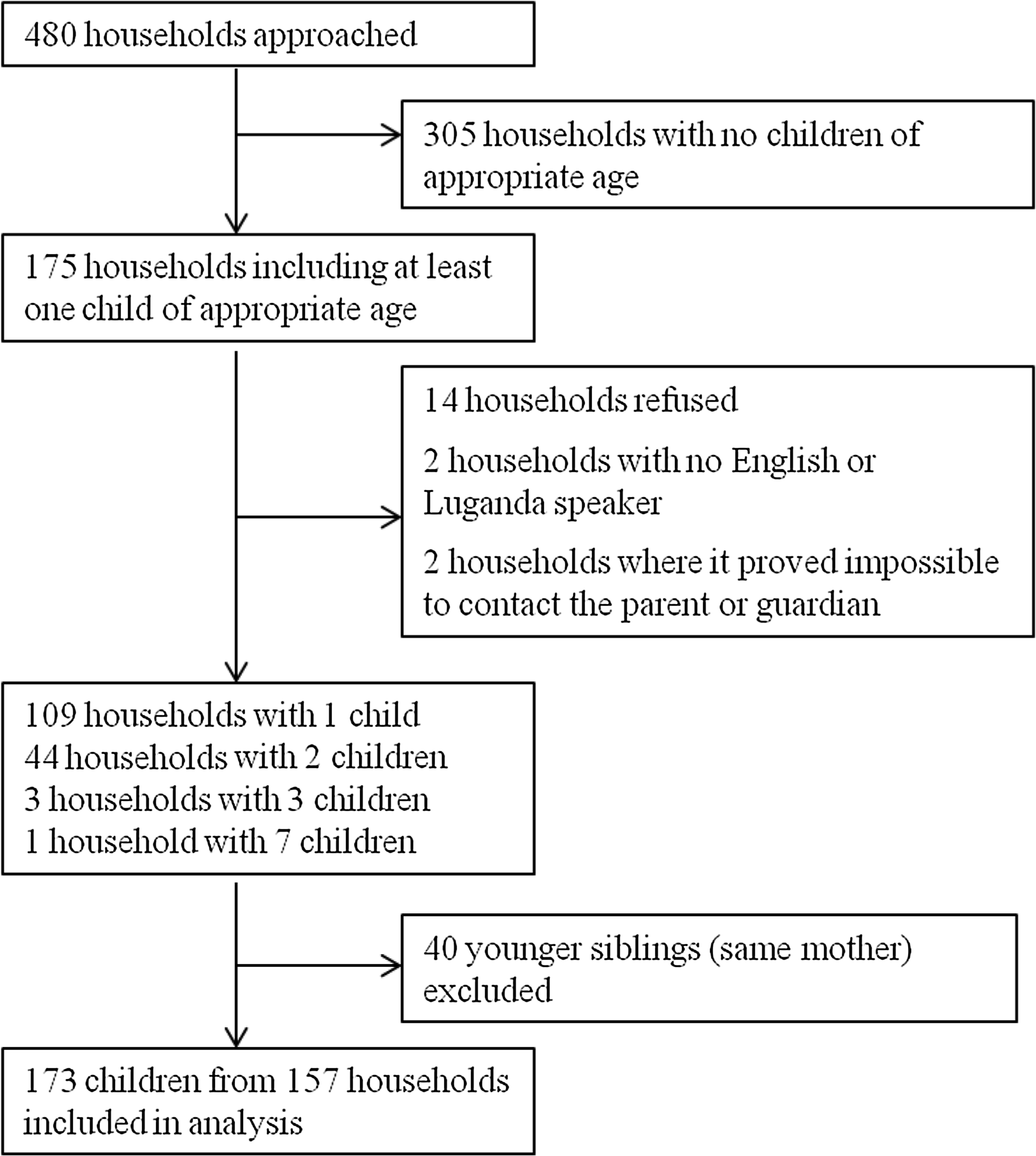

Supplement: Supplementary file 1 — Authors’ original file for figure 1 [file 13063_2014_2191_MOESM1_ESM.tif]

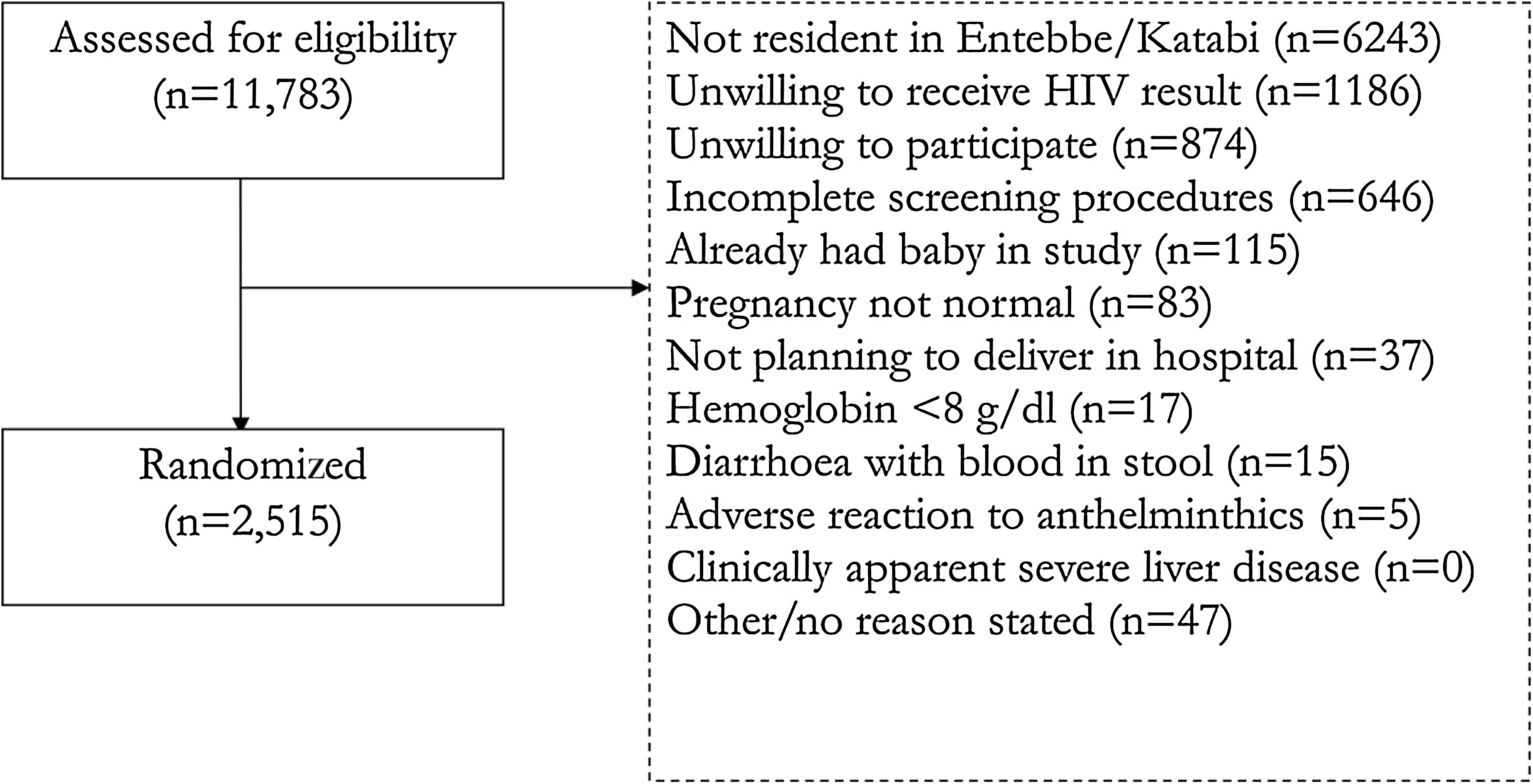

Supplement: Supplementary file 2 — Authors’ original file for figure 2 [file 13063_2014_2191_MOESM2_ESM.tif]
